# Anti-inflammatory effects of antidepressant and atypical antipsychotic medication for the treatment of major depression and comorbid arthritis: a case report

**DOI:** 10.1186/1752-1947-4-6

**Published:** 2010-01-12

**Authors:** Bernhard T Baune, Harris Eyre

**Affiliations:** 1 Psychiatry and Psychiatric Neuroscience, School of Medicine and Dentistry, James Cook University, Townsville 4811, Australia

## Abstract

**Introduction:**

This case report describes the effects of psychotropic treatment, quetiapine in particular, on systemic inflammation, pain, general functioning and major depression in the treatment of a woman with arthritis.

**Case presentation:**

A 49-year-old Caucasian Australian woman with arthritis, pain and depression was treated with a course of escitalopram, mirtazapine and quetiapine. Pain levels, general functioning and degree of depressive symptoms were evaluated with a visual analogue scale. Systemic inflammation had been assessed by C-reactive protein serum levels since 2003. C-reactive protein levels, physical pain, symptoms of arthritis and depression decreased significantly during the past 12 months of treatment with quetiapine, while treatment with selective serotonin reuptake inhibitors and mirtazapine remained the same.

**Conclusions:**

We suggest that the treatment particularly with quetiapine may have anti-inflammatory effects in arthritis and comorbid major depression, which eventually led to a remission of pain and depression and to normal general function.

## Introduction

Patients with major depression often suffer comorbid physical disorders [1] of which some are related to systemic inflammation, such as cardiovascular disease [2] and rheumatoid arthritis [3]. In turn, systemic inflammation has been related to the onset and course of depression [4]. It has been suggested that stress and inflammatory pathways are involved in the response to antidepressant treatment [5].

In this case report, we describe a patient with chronic psoriatic arthritis that cause impairing pain, increased C-reactive protein levels (CRP) and depressive symptoms. However, a significant improvement of arthritic symptoms including pain and mental symptoms, as well as a decrease in CRP, was observed after the patient was commenced on a combined treatment of psychotropic medication (antidepressant and atypical neuroleptic). The treatment subsequently led to a significant increase in the patient's levels of general functioning.

## Case presentation

A 49-year-old Caucasian Australian woman was referred by her general practitioner to a specialist clinic for mood disorders in October 2007 with the request to assess and manage longstanding symptoms of depression. Her history shows psoriatic arthritis causing significant pain since age 43 and mild to moderate depressive episodes since her early 30 s. In October 2007, the patient presented with severe emotional disturbance characterised by anxiety, frustration and depression. These feelings seemed to be largely brought about by her decreased mobility as a result of severe, debilitating arthritic pain in her joints that worsened over the past 12 months prior to assessment. Due to her depression which had started in 2002, she was seen by her psychiatrist in the community, who diagnosed her with major depressive disorder (MDD).

Since the diagnosis of psoriatic arthritis in 2002 until mid-2007, the patient's arthritic symptom profile had been progressively worsening. Concomitantly, her depressive symptoms were worsening over this period of time due partly to a personal experience of loss (death of her mother).

**Figure 1:**
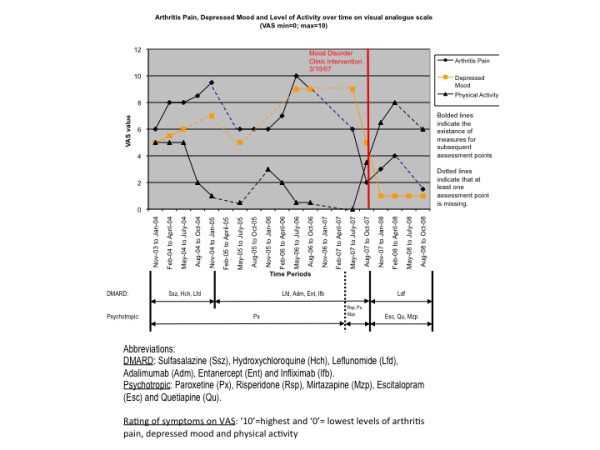
**Development of Arthritis Pain, Depressed Mood and Level of Activity over time**. The graph describes the course of levels of arthritis pain, depressive symptoms and physical activity over an extended period between Nov. 2003 and Oct. 2008 depending on DMARD and psychotropic medication. The psychiatric intervention in Oct. 2007 through the mood disorder clinic and the subsequent change in DMARD and psychotropic medication indicates a significant change in symptom presentation with a decline in pain and depressive symptoms while the activity levels increased continuously.

When her arthritic disease was worsening between early 2005 and 2007, she was started on a number of disease-modifying antirheumatic drugs (DMARDs) and analgesics (oxycodone, tramadol and paracetamol combined with codeine and taken regularly since 2002) as prescribed by her rheumatologist (Figure 1). Her intake of DMARDs was as follows: sulfasalazine (500 mg BID), hydroxychloroquine (200 mg BD) and leflunomide (20 mg QD) from November 2003 to November 2004; leflunomide (20 mg QD), adalimumab (40 mg once at night), etanercept (50 mg SC weekly) and infliximab (300 mg/infusion) from December 2004 to October 2007; and then only leflunomide (20 mg QD) since October 2007.

Due to a significant aggravation of her depressive mood, mood instability, poor sleep and a decrease in her general functioning, she was treated for MDD with paroxetine 20 mg/mane in 2002, with risperidone 0.5 mg/bd in 2007, and then with mirtazapine up to 45 mg/nocte two months before she was assessed for the first time in a mood disorder clinic in October 2007.

Based on her psychiatric assessment in October 2007, the previous diagnosis of MDD was confirmed and she was started on a new course of treatment. While staying on the same DMARD (leflunomide, 20 mg QD) from October 2007 onwards, she was commenced on courses of escitalopram 10 mg/mane (replacing paroxetine), quetiapine 50 mg/mane and 100 mg/nocte (replacing risperidone, which was previously prescribed to improve worried thought content), and continued on mirtazapine 30 mg/nocte. She was commenced on quetiapine since recent evidence suggests the effectiveness of quetiapine in major depression [6].

In the follow-up consultations in the mood disorder clinic it became apparent that her depressive symptoms were continuously improving, and following a month lag phase her arthritic pain decreased significantly, her mobility increased, and her CRP levels decreased. This resulted in a drastically improved depression, which eventually led to a remission after three months of treatment.

Between the commencement of the new psychotropic treatment regimen in October 2007 and the significant improvement of the depressive symptoms as described above, the arthritis specific treatment (DMARDs and analgesics) had not changed. While her CRP levels were 32 mg/L before the start of the new psychotropic treatment regimen, they dropped continuously to 13 mg/L over 10 months between October 2007 and July 2008 without changing the arthritis specific medication. Due to the significant decrease in her pain levels, depressive symptoms and CRP levels, the anti-tumor necrosis factor treatment (abatcept) discussed previously by her rheumatologist was no longer considered as a necessary treatment option. In addition, the patient did not have any significant lifestyle changes during the same period of time.

While staying on the above psychotropic medication, the patient was able to maintain a low level of arthritic pain throughout the year 2008. Despite some relatively mild intermittent increase of some of her arthritic pain and depressive symptoms, which were interpreted as relatively mild fluctuations over a 12-month period, her level of general functioning remained high.

## Discussion

This case report has a number of interesting findings that warrant further exploration and discussion. First, the patient's depression symptoms were timely and closely related to physical symptoms such as pain from the arthritis. Secondly, the combination of selective serotonin reuptake inhibitors (SSRI), antidepressants and quetiapine as atypical antipsychotic treatment lifted the patient's depression, physical symptoms and CRP levels. Lastly, her previous treatment with a combination of drugs of similar classes failed to neither produce relevant relief of either mental and/or physical symptoms nor lower her CRP levels.

Previous studies found that depression is more common in arthritis than in healthy subjects [7] and both disorders are closely related [8]. Depression and arthritis may be further linked through common inflammatory pathways, in which cytokines seem to play a major role [8]. This case report is supports the suggestion that joint inflammatory pathways between arthritis and depression as symptoms of arthritis, pain and depression are simultaneously lifted when CRP levels are lowered.

Although there are some conflicting results, it has been suggested that antidepressants can lower the levels of systemic inflammation markers, such as CRP [9] and cytokines, while also having analgesic effects [10,11]. A few experiments suggest that paroxetine [12], fluoxetine and clomipramine [10] also have anti-inflammatory effects. Extensive research has not been carried out in this area, thus leaving a lack of data on the potential anti-inflammatory effects of escitalopram despite a recent finding that escitalopram lowers the level of soluble interleukin 2 receptor [13]. However, since the patient was treated with SSRI antidepressants before (paroxetine) and after (escitalopram), it can be suggested that the large clinical improvement and reduction in her CRP was not related to the SSRI treatment alone. Moreover, the findings in this case report are less likely to be influenced by the effects of mirtazapine as the patient had been treated continuously with this compound long before and after the clinical improvement.

This leaves us to the discussion of the anti-inflammatory effects of antipsychotic medication on CRP levels and a patient's clinical symptoms. The literature shows less inconsistent findings on the potential anti-inflammatory effects of antipsychotics. While some reports indicate that patients treated with antipsychotics such as olanzapine exhibit higher levels of inflammatory markers including CRP [14,15], some early evidence suggests that clozapine may also decrease inflammatory response as evinced by a parallel decrease in cytokine expression [16].

In addition, some atypical antipsychotics such as risperidone are thought to exert a positive effect on the inflammatory response system in patients with schizophrenia, which possibly accounts for a better treatment outcome as compared to conventional neuroleptics [17]. More particularly, risperidone has been shown to reduce pro-inflammatory cytokines such as tumor necrosis factor alpha and interleukin 6 (IL-6) in mice treated with lipopolysaccharide (LPS) [18]. In this case report, the treatment with risperidone failed to show reductions in CRP level and relevant symptoms of arthritis, pain and depression. In contrast, our patient's commencement on quetiapine treatment, while her SSRI medication was maintained, was related to a reduction in her CRP levels and a clinical improvement in all other areas.

Since no reports on the possible anti-inflammatory effects, including effects on CRP levels, of quetiapine have been published, our findings indicate that the antipsychotic drug quetiapine may have exerted such anti-inflammatory effects. In addition, the sleep-inducing properties of quetiapine might have contributed to a symptom relief through the link between sleep improvement and pain relief. All together, our patient's commencement on the antipsychotic medication quetiapine is possibly responsible for the significant clinical improvement in her physical and mental symptoms and for the decline in her CRP levels.

## Conclusions

This case report suggests that in addition to the known anti-inflammatory effects of SSRI, the treatment particularly with quetipiane may have stronger anti-inflammatory (reduction of CRP levels) effects in arthritis and comorbid major depression, which yielded a clinical improvement of pain, depression and general function.

## Abbreviations

CRP: C-reactive protein; DMARD: disease-modifying antirheumatic drug; LPS: lipopolysaccharide; MDD: major depressive disorder; SSRI: selective serotonin reuptake inhibitors.

## Consent

Written informed consent was obtained from the patient for publication of this case report and any accompanying images. A copy of the written consent is available for review by the Editor-in-Chief of this journal.

## Competing interests

The authors declare that they have no competing interests.

## Authors' contributions

BB served as the patient's psychiatrist. He also drafted the manuscript. HE compiled clinical data, obtained additional clinical information from the patient. He also created the figures cited in this case report. Both authors read and approved the final manuscript.
